# Role of the Protein Tyrosine Phosphatase Shp2 in Homeostasis of the Intestinal Epithelium

**DOI:** 10.1371/journal.pone.0092904

**Published:** 2014-03-27

**Authors:** Hironori Yamashita, Takenori Kotani, Jung-ha Park, Yoji Murata, Hideki Okazawa, Hiroshi Ohnishi, Yonson Ku, Takashi Matozaki

**Affiliations:** 1 Division of Molecular and Cellular Signaling, Department of Biochemistry and Molecular Biology, Kobe University Graduate School of Medicine, Kobe, Hyogo, Japan; 2 Division of Hepato-Biliary-Pancreatic Surgery, Department of Surgery, Kobe University Graduate School of Medicine, Kobe, Hyogo, Japan; 3 Department of Laboratory Sciences, Gunma University Graduate School of Health Sciences, Maebashi, Gunma, Japan; 4 Laboratory of Biosignal Sciences, Institute for Molecular and Cellular Regulation, Gunma University, Maebashi, Gunma, Japan; Kinki University School of Pharmaceutical Sciences, Japan

## Abstract

Protein tyrosine phosphorylation is thought to be important for regulation of the proliferation, differentiation, and rapid turnover of intestinal epithelial cells (IECs). The role of protein tyrosine phosphatases in such homeostatic regulation of IECs has remained largely unknown, however. Src homology 2–containing protein tyrosine phosphatase (Shp2) is a ubiquitously expressed cytoplasmic protein tyrosine phosphatase that functions as a positive regulator of the Ras–mitogen-activated protein kinase (MAPK) signaling pathway operative downstream of the receptors for various growth factors and cytokines, and it is thereby thought to contribute to the regulation of cell proliferation and differentiation. We now show that mice lacking Shp2 specifically in IECs (Shp2 CKO mice) develop severe colitis and die as early as 3 to 4 weeks after birth. The number of goblet cells in both the small intestine and colon of Shp2 CKO mice was markedly reduced compared with that for control mice. Furthermore, Shp2 CKO mice showed marked impairment of both IEC migration along the crypt-villus axis in the small intestine and the development of intestinal organoids from isolated crypts. The colitis as well as the reduction in the number of goblet cells apparent in Shp2 CKO mice were normalized by expression of an activated form of K-Ras in IECs. Our results thus suggest that Shp2 regulates IEC homeostasis through activation of Ras and thereby protects against the development of colitis.

## Introduction

Intestinal epithelial cells (IECs) play a central role in the absorption of nutrients and water by the intestine as well as contribute to protection against ingested pathogens by providing a functional barrier. In mammals, IECs of the small and large intestine are regenerated continuously from stem cells throughout adulthood [1]. The stem cells that give rise to IECs reside in a region near the base of intestinal crypts. These stem cells generate proliferating progeny, known as transient amplifying (TA) cells, that migrate out of the stem cell niche, cease to proliferate, and initiate differentiation into the various cell lineages of mature intestinal villi, including absorptive enterocytes, mucin-secreting goblet cells, peptide hormone–secreting neuroendocrine cells, and antimicrobial peptide–producing Paneth cells [2]. Cells of the first three of these four lineages mature and migrate up the crypt toward the tip of intestinal villi, whereas Paneth cells travel down to the base of the crypt. Absorptive enterocytes in particular have a short life span, being released into the gut lumen after they have migrated to the tip of the villi. Such elimination of IECs is thought to be triggered by either spontaneous apoptosis [3] or overcrowding [4] of IECs, although the mechanism by which the precise timing of elimination is determined remains poorly understood. The continuous production of new IECs from each crypt is thus balanced by the elimination of older cells at the luminal side of the intestine, resulting in a rapid turnover of IECs (4 to 5 days in the mouse) [1], [2].

Protein tyrosine phosphorylation is an important signaling mechanism that regulates the proliferation, differentiation, migration, and survival of IECs. For instance, epidermal growth factor (EGF), whose receptor is a protein tyrosine kinase (PTK), is thought to be essential for IEC proliferation [5]. In addition, ephrins and their PTK receptors are implicated in the proliferation of intestinal stem cells and the positioning of IECs along the crypt-villus axis [1], [6], [7]. In contrast to PTKs, the importance of protein tyrosine phosphatases (PTPs) in the regulation of IECs has remained largely unknown, with the exception that stomach cancer–associated protein tyrosine phosphatase–1 (SAP-1, also known as PTPRH), a receptor-type PTP, is specifically expressed in IECs [8] and might play a role in control of the proliferation or migration of these cells [9].

Src homology 2–containing protein tyrosine phosphatase 2 (Shp2, also known as PTPN11) is a cytoplasmic PTP that contains two tandem Src homology 2 (SH2) domains [10], [11]. Shp2 is expressed in most mammalian cell types and is thought to bind through its SH2 domains to the tyrosine-phosphorylated platelet-derived growth factor (PDGF) receptor as well as to tyrosine-phosphorylated docking proteins (such as insulin receptor substrates, signal regulatory protein α, and Grb2-associated binder proteins) in response to cell stimulation with growth factors or to cell adhesion. Such binding is important both for activation of the PTP activity of Shp2 as well as for its recruitment to sites near the plasma membrane where potential substrates are located [10], [11]. Although PTPs are generally considered to be negative regulators on the basis of their ability to oppose the effects of PTKs, biochemical and genetic analyses indicate that Shp2 is required for activation of the Ras–mitogen-activated protein kinase (MAPK) signaling pathway operative downstream of the receptors for various growth factors and cytokines, and that it thereby contributes to the promotion of cell proliferation, differentiation, or survival [10], [11]. Moreover, Shp2 is also implicated in the regulation of cell adhesion and migration, in part through its control of the activity of the small GTP-binding protein Rho [12], [13]. Indeed, homozygous Shp2 mutant mice were found to die as embryos as a result of a defect in gastrulation and abnormal mesoderm patterning [14].

The precise role of Shp2 in the regulation of IEC function, especially in vivo, has remained unclear, however. To address this issue, we have now generated and analyzed IEC-specific Shp2 conditional knockout (CKO) mice.

## Materials and Methods

### Ethics Statement

This study was approved by the Institutional Animal Care and Use Committee of Kobe University (Permit Number: P130206, P120304-R2, P110402), and all animal experiments were performed according to Kobe University Animal Experimentation Regulations. All efforts were made to minimize suffering.

### Antibodies and reagents

A mouse monoclonal antibody (mAb) to β-catenin was obtained from BD Biosciences (San Diego, CA), a mouse mAb to β-tubulin was from Sigma-Aldrich (St. Louis, MO), and a rat mAb to bromodeoxyuridine (BrdU) was from Abcam (Cambridge, MA). Rabbit polyclonal antibodies (pAbs) to Shp2 and to mucin 2 were obtained from Santa Cruz Biotechnology (Santa Cruz, CA), those to lysozyme were from Dako (Glostrup, Denmark), and those to cleaved caspase-3 (Asp^175^) were from Cell Signaling Technology (Danvers, MA). Alkaline phosphatase–conjugated sheep pAbs to digoxigenin for in situ hybridization were obtained from Roche (Basel, Swizerland). Cy3- or Alexa Fluor 488–conjugated goat secondary pAbs for immunofluorescence analysis were obtained from Jackson ImmunoResearch (West Grove, PA) and Invitrogen (Carlsbad, CA), respectively, and 4',6-diamino-2-phenylindole (DAPI) was from Nacalai Tesque (Kyoto, Japan). Horseradish peroxidase–conjugated goat secondary pAbs for immunoblot analysis were obtained from Jackson ImmunoResearch. Mayer's hemalum solution was from Merck KGaA (Darmstadt, Germany), and eosin was from Wako (Osaka, Japan).

### Mice

*Ptpn11* ^fl/fl^ mice were kindly provided by Dr. B. G. Neel (Princess Margaret Cancer Centre) [15]. The Rosa26 conditional reporter strain (*R26R*) of mice (B6.129S4-*Gt(ROSA)26Sor^tm1Sor^*/J) [16], *LSL-Kras G12D* mice (B6.129S4-*Kras^tm4Tyj^*/J) [17], and villin-*cre* mice (B6.SJL-Tg(Vil-cre)997Gum/J) [18] were obtained from Jackson Laboratory (Bar Harbor, ME). *R26R*;villin-*cre* mice were obtained by crossing *R26R* mice with villin-*cre* mice. To generate *Ptpn11* ^fl/+^;villin-*cre* mice, we crossed villin-*cre* mice with *Ptpn11* ^fl/fl^ mice. The resulting *Ptpn11* ^fl/+^;villin-*cre* offspring were crossed with *Ptpn11* ^fl/fl^ mice to obtain *Ptpn11* ^fl/fl^;villin-*cre* (Shp2 CKO) mice and *Ptpn11* ^fl/fl^ (control) mice. To generate *Ptpn11* ^fl/+^;villin*-cre;LSL-Kras G12D* mice, we crossed Shp2 CKO mice with *LSL-Kras G12D* mice. The resulting *Ptpn11* ^fl/+^;villin-*cre;LSL-Kras G12D* offspring were crossed with *Ptpn11* ^fl/fl^ mice to obtain *Ptpn11* ^fl/fl^;villin*-cre;LSL-Kras G12D* (Shp2 CKO;*LSL-Kras G12D*) mice and Shp2 CKO mice. All mice were maintained in the Institute for Experimental Animals at Kobe University Graduate School of Medicine under specific pathogen–free conditions. The genotype of all offspring was determined by polymerase chain reaction (PCR) analysis.

### Detection of deleted and floxed alleles of Ptpn11 by PCR

For preparation of genomic DNA, various tissues isolated from adult control or Shp2 CKO mice were washed with ice-cold phosphate-buffered saline (PBS), incubated overnight at 56°C in lysis buffer (100 mM Tris-HCl [pH 8.5], 5 mM EDTA, 0.2% SDS, 200 mM NaCl, proteinase K [50 μg/ml]), and centrifuged at 17,500×*g* for 15 min at 4°C. The resulting supernatant was subjected to isopropanol precipitation for separation of genomic DNA. The floxed *Ptpn11* allele (∼400-bp product) was identified by PCR with the sense primer SHP2F (5'-TAGCTGCTTTAACCCTCTGTGT-3') and the antisense primer SHP2R (5'-CATCAGAGCAGGCCATATTCC-3'), whereas the deleted allele (∼500-bp product) was identified with the sense primer SHP2F and the antisense primer SHP2R#3 (5'-TCACAATGAAGGTTCCTGTCC-3').

### Isolation of mouse IECs

Mouse IECs were isolated as previously described [19] but with slight modifications. In brief, the freshly isolated whole intestine of adult control or Shp2 CKO mice was washed with PBS, cut into small pieces, washed three times with Hanks' balanced salt solution (HBSS) containing 1% fetal bovine serum and 25 mM HEPES-NaOH (pH 7.5), and then incubated three or four times on a rolling platform for 15 min at room temperature in HBSS containing 50 mM EDTA and 25 mM HEPES-NaOH (pH 7.5). The tissue debris was removed, and IECs in the resulting supernatant were isolated by centrifugation at 250×*g* for 10 min at 4°C and washed three times with PBS.

### Immunoblot analysis

Isolated cells or tissues were washed with ice-cold PBS and then homogenized on ice in RIPA buffer (20 mM Tris-HCl [pH 7.5], 150 mM NaCl, 2 mM EDTA, 1% Nonidet P-40, 1% sodium deoxycholate, 0.1% SDS, 50 mM NaF) containing 1 mM sodium vanadate and a protease inhibitor cocktail (Nacalai Tesque). The lysates were centrifuged at 17,500×*g* for 15 min at 4°C, and the resulting supernatants were subjected to immunoblot analysis as previously described [8], [19].

### Assessment of colitis

For assessment of colitis, Shp2 CKO and control mice were weighed weekly and monitored for the appearance of diarrhea, blood in the stool, and anorectal prolapse. Disease activity was scored as previously described [20], [21] with minor modifications. Stool consistency was scored as: 0 = normal, 2 = loose stools, 4 = liquid stools. Blood in the stool was scored as: 0 = no blood as revealed with the guaiac occult blood test (Occult Blood Slide II; Shionogi Pharmaceutical, Osaka, Japan), 2 = positive guaiac occult blood test, 4 = gross bleeding. Development of anorectal prolapse was scored as: 0 = no prolapse, 2 = prolapse evident only during defecation, 4 = prolapse evident at all times. The total score for diarrhea, blood in the stool, and prolapse, ranging from 0 (normal) to 12 (severe), was determined as the disease activity index (DAI).

### Histology and immunofluorescence analysis

For histological analysis, the small intestine and colon were removed and immediately fixed for 3 h at room temperature with 4% paraformaldehyde in PBS. Paraffin-embedded sections (thickness of 5 μm) were then prepared and stained with hematoxylin-eosin. For immunofluorescence analysis, the small intestine or colon was fixed as for histology and then transferred to a series of sucrose solutions (7, 20, and 30% [w/v], sequentially) in PBS for cryoprotection, embedded in OCT compound (Sakura, Tokyo, Japan), and rapidly frozen in liquid nitrogen. Frozen sections with a thickness of 5 μm were prepared with a cryostat, mounted on glass slides, and air-dried. The sections were then subjected to immunofluorescence analysis with primary antibodies and fluorescent dye–labeled secondary antibodies as described previously [8]. Images were obtained with a fluorescence microscope (BX51; Olympus, Tokyo, Japan).

### β-Galactosidase staining

Staining for β-galactosidase was performed as previously described [22] with slight modifications. In brief, the colon and small intestine were removed, fixed for 30 min at 4°C with 0.2% glutaraldehyde and 4% paraformaldehyde in PBS, and washed with PBS. The tissue was then transferred to a series of sucrose solutions (7, 20, and 30% [w/v], sequentially) in PBS, embedded in OCT compound, and rapidly frozen with liquid nitrogen. Frozen sections with a thickness of 10 μm were prepared and then stained for 2 to 10 h at 37°C with β-galactosidase substrate (X-gal [1 mg/ml], 4 mM K_3_Fe(CN)_6_, 4 mM K_4_Fe(CN)_6_-3H_2_O, 2 mM MgCl_2_) in PBS. Stained sections were examined with a fluorescence microscope (BX51, Olympus).

### In situ hybridization

Expression of the Olfactomedin4 (Olfm4) gene in the intestinal epithelium was examined by in situ hybridization performed as described previously [23]. In brief, paraffin-embedded sections of the ileum (thickness of 10 μm) were depleted of paraffin with xylene, rehydrated by exposure to a graded series of ethanol solutions, and treated with 0.2 M HCl and proteinase K. The sections were then fixed again with 4% paraformaldehyde, demethylated with acetic anhydride, and subjected to hybridization for 48 h at 65°C with a digoxigenin-labeled RNA probe for Olfm4 mRNA (IMAGE clone 1078130) at 500 ng/ml. They were then incubated overnight at 4°C with alkaline phosphatase–conjugated pAbs to digoxigenin, washed, and incubated with nitro blue tetrazolium chloride and 5-bromo-4-chloro-3-indolyl phosphate (Sigma-Aldrich). Images were obtained with a fluorescence microscope (BX51, Olympus).

### BrdU incorporation assay

Mice were injected intraperitoneally with BrdU (50 mg/kg) and killed 2 or 48 h later. The ileum or colon was fixed with 4% paraformaldehyde, transferred to a series of sucrose solutions in PBS, embedded in OCT compound, and rapidly frozen with liquid nitrogen as described for immunofluorescence analysis. Sections with a thickness of 5 μm were incubated for 30 min at 65°C with 0.025 M HCl, washed with 0.1 M borate buffer (pH 8.5), and incubated at room temperature first for 2 h with mAbs to BrdU and to β-catenin and then for 1 h at with fluorescent dye–labeled secondary pAbs. Fluorescence images were obtained with a fluorescence microscope (BX51, Olympus). IEC migration distance was defined as the distance from the crypt base to the farthest migrated BrdU-positive cells and was measured with the use of ImageJ software (NIH).

### Intestinal organoid culture

Intestinal organoid culture was performed as previously described [5]. In brief, crypts were isolated from the small intestine by incubation for 30 min in PBS containing 2 mM EDTA. The isolated crypts were mixed with Matrigel (BD Biosciences) and transferred to 48-well plates. After polymerization of the Matrigel, advanced Dulbecco's modified Eagle's medium–F12 (Invitrogen), which was supplemented with penicillin-streptomycin (100 U/ml) (Invitrogen), 10 mM HEPES (Invitrogen), 1× GlutaMAX (Invitrogen), 1× N2 (Invitrogen), 1× B27 (Invitrogen), 1.25 mM *N*-acetylcysteine (Sigma-Aldrich), epidermal growth factor (500 ng/ml) (Peprotech, Rocky Hill, NJ), 10% R-spondin1–Fc–conditioned medium, and Noggin (100 ng/ml) (Peprotech), was overlaid on the gel in each well. The cultures were then maintained in an incubator (37°C, 5% CO_2_). Images were obtained with a microscope (Axiovert 200; Carl Zeiss, Oberkochen, Germany).

### Statistical analysis

Disease activity was compared between mouse genotypes with the Wilcoxon rank-sum test. Other quantitative data are presented as means ± SE and were analyzed with Student's *t* test, the Wilcoxon rank-sum test, or Welch's *t* test as appropriate. All statistical analysis was performed with JMP software version 9.0 (SAS Institute, Cary, NC). A *P* value of <0.05 was considered statistically significant.

## Results

### Generation of IEC-specific Shp2 CKO mice

**Figure 1:**
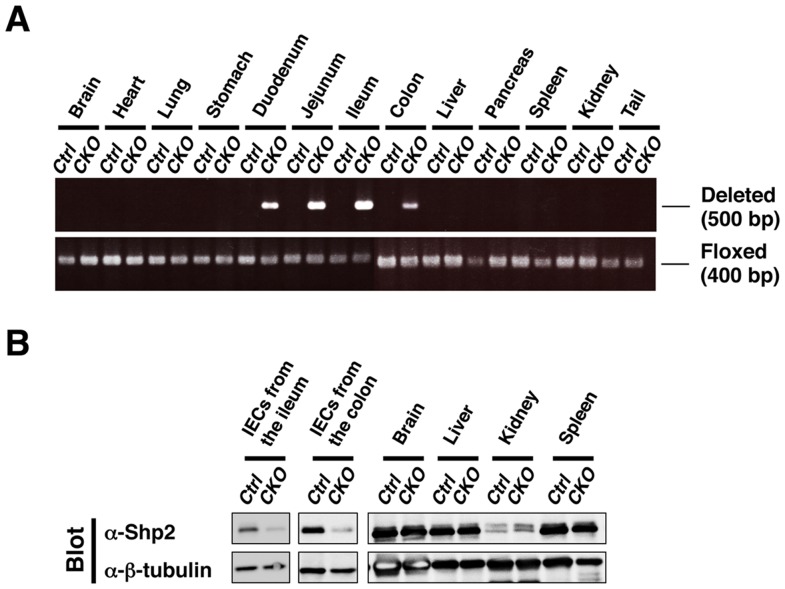
Generation of IEC-specific Shp2 CKO mice. **A**: Genomic DNA extracted from the indicated organs of adult control (Ctrl) or Shp2 CKO (CKO) mice was subjected to PCR analysis with primers specific for deleted or floxed alleles of *Ptpn11*. Data are representative of three separate experiments. **B**: Lysates of IECs (from the ileum or colon) and the indicated organs from control or Shp2 CKO mice were subjected to immunoblot analysis with antibodies to (α-) both Shp2 and β-tubulin (loading control). Data are representative of three (IECs) or two (indicated organs) separate experiments.

To examine the impact of Shp2 ablation in IECs, we crossed mice homozygous for a floxed *Ptpn11* allele [15] with those harboring a transgene for Cre recombinase under the control of the villin gene promoter (villin-*cre*) [18]. The specificity and efficiency of *Ptpn11* deletion in adult *Ptpn11* ^fl/fl^;villin-*cre* (Shp2 CKO) mice were determined by PCR analysis of genomic DNA isolated from the intestine as well as from other organs. Consistent with the results of previous studies with the villin-*cre* transgene [18], deleted *Ptpn11* alleles were detected in the colon, ileum, jejunum, and duodenum of Shp2 CKO mice, but not in any other organ (**Fig. 1A**). We also confirmed that the activity of β-galactosidase was detected specifically in the entire epithelium of the ileum and colon of *R26R*;villin-*cre* mice (**Fig. S1**). Immunoblot analysis also showed that the abundance of Shp2 protein in isolated IECs from the ileum or colon of Shp2 CKO mice was greatly reduced compared with that for control mice, whereas it was unaffected in other organs (**Fig. 1B**). These results thus indicated that the villin-*cre* transgene directs the efficient and specific deletion of the Shp2 gene in IECs.

### Shp2 CKO mice develop severe colitis

**Figure 2:**
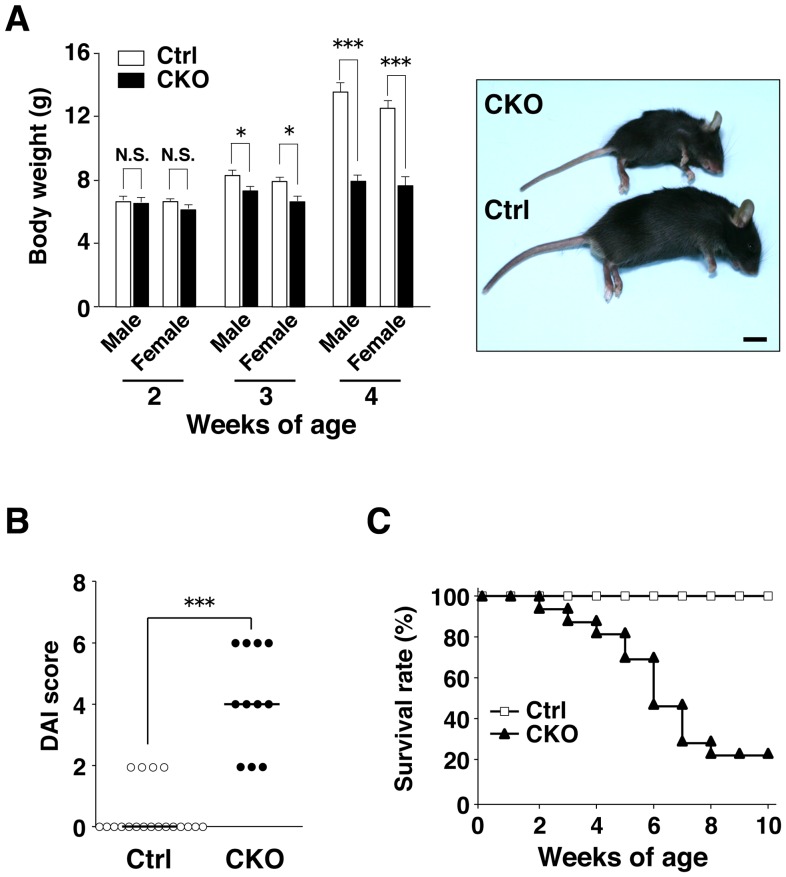
Shp2 CKO mice develop severe colitis. **A**: Body weight of 2-, 3-, or 4-week-old control (male, *n* = 23, 26, and 27, respectively; female, *n* = 22, 24, and 22, respectively) or Shp2 CKO (male, *n* = 17, 20, and 16, respectively; female, *n* = 15, 15, and 13, respectively) mice is shown in the left panel. Data are means ± SE. *P<0.05, ***P<0.0001 (Welch's *t* test). N.S., not significant. Representative photographs of control and Shp2 CKO mice at 5 weeks of age are shown in the right panel. Scale bar, 1 cm. **B**: Disease activity index (DAI) for colitis in control (*n* = 19) and Shp2 CKO (*n* = 11) mice at 4 to 5 weeks of age. Bars indicate median scores. ***P<0.0001 (Wilcoxon rank-sum test). **C**: Survival rates of control (*n* = 19) and Shp2 CKO (*n* = 17) mice.

Shp2 CKO mice were born apparently healthy (data not shown), and they were phenotypically indistinguishable from control littermates at 2 weeks of age (**Fig. 2A**). However, both male and female Shp2 CKO mice manifested a marked reduction in body weight as well as growth retardation compared with control littermates at 3 to 4 weeks of age (**Fig. 2A**). They also manifested severe diarrhea. We evaluated disease activity for colitis on the basis of stool consistency, blood in the stool, and anorectal prolapse in both Shp2 CKO and control mice at 4 to 5 weeks of age. The disease activity index for Shp2 CKO mice was markedly greater than that for control animals (**Fig. 2B**). Furthermore, ∼80% of Shp2 CKO mice died by 10 weeks of age (**Fig. 2C**).

**Figure 3:**
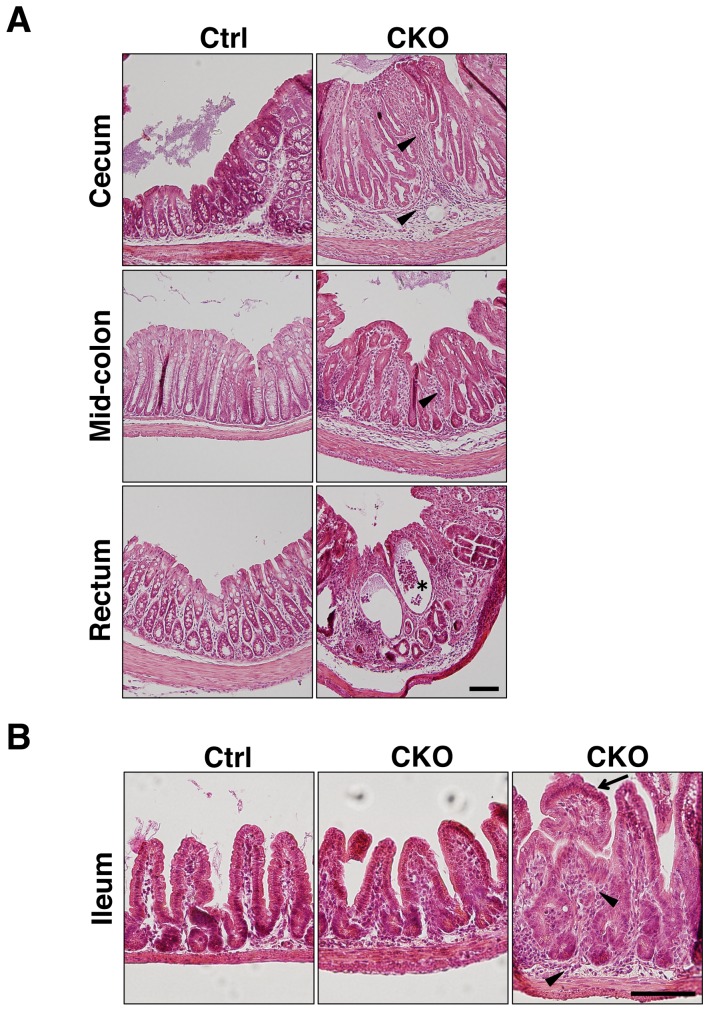
Spontaneous development of marked inflammation in the colon of Shp2 CKO mice. **A**: Hematoxylin-eosin staining of paraffin-embedded sections of the cecum, mid-colon, and rectum from control or Shp2 CKO mice at 3 weeks of age. Arrowheads and the asterisk indicate inflammatory infiltrates and a crypt abscess, respectively. **B**: Hematoxylin-eosin staining of the ileum from control and Shp2 CKO mice at 3 weeks of age. Arrowheads and the arrow indicate inflammatory infiltrates and abnormal villus structure, respectively. All data are representative of three separate experiments. Scale bars, 100 μm.

Histological examination of the colon from 3-week-old Shp2 CKO mice revealed pronounced inflammation in all regions from the cecum to the rectum (**Fig. 3A**). Epithelial hyperplasia was relatively prominent, and transmural inflammation with crypt abscesses was occasionally observed. Inflammatory infiltrates were also present in both the mucosa and submucosa. In contrast to the colon, most regions of the small intestine of Shp2 CKO mice were phenotypically indistinguishable from those of control mice, although abnormal structures of villi and mild inflammatory infiltrates were occasionally apparent. Together, these observations indicated that Shp2 ablation in IECs resulted in the spontaneous development of marked inflammation in the intestine, particularly in the colon.

### Marked reduction in the numbers of absorptive enterocytes and goblet cells in Shp2 CKO mice

**Figure 4:**
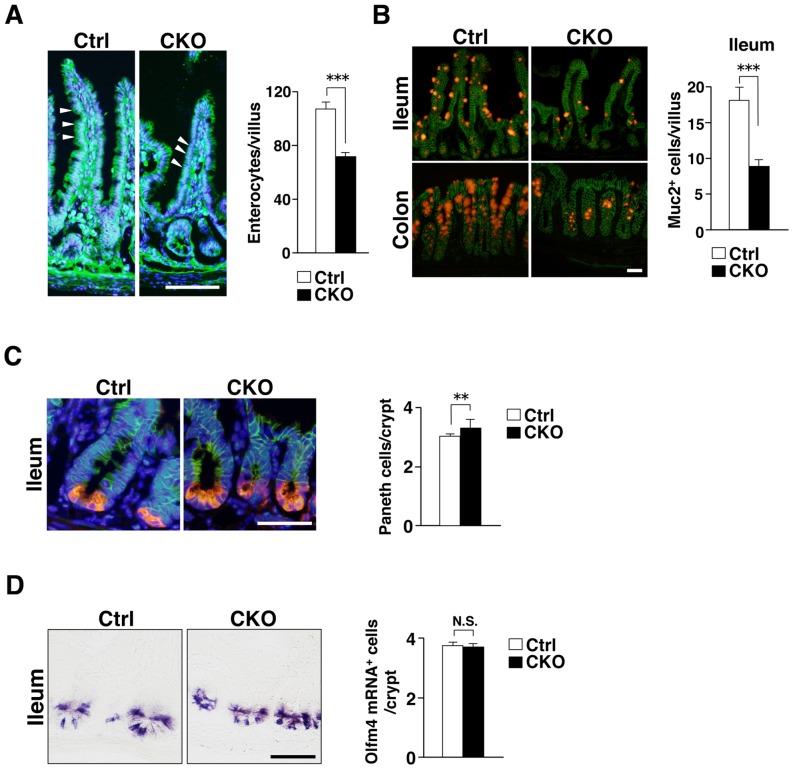
Marked reduction in the numbers of absorptive enterocytes and goblet cells in Shp2 CKO mice. **A**: Frozen sections of the ileum from control or Shp2 CKO mice at 3 to 4 weeks of age were immunostained with an antibody to β-catenin (green) and also stained with DAPI (blue). Representative images are shown in the left panel. Arrowheads indicate β-catenin–positive absorptive enterocytes. Scale bar, 100 μm. The number of β-catenin–positive absorptive enterocytes per villus was determined for the ileum (right panel). Data are means ± SE for 38 (control) or 32 (Shp2 CKO) villi from a total of three mice per group. ***P<0.0001 (Welch's *t* test). **B**: Frozen sections of the ileum and colon from control or Shp2 CKO mice at 3 weeks of age were immunostained with antibodies to mucin 2 (red) and to β-catenin (green). Representative images are shown in the left panel. Scale bar, 100 μm. The number of mucin 2 (Muc2)–positive goblet cells per villus was determined for the ileum (right panel). Data are means ± SE for 100 (control) or 93 (Shp2 CKO) villi from a total of three mice per group. ***P<0.0001 (Welch's *t* test). **C**: Frozen sections of the ileum from control or Shp2 CKO mice at 3 weeks of age were subjected to immunostaining with antibodies to lysozyme (red) and to β-catenin (green) as well as to staining of nuclei with DAPI (blue). Representative images are shown in the left panel. Scale bar, 50 μm. The number of lysozyme-positive Paneth cells per crypt was determined (right panel). Data are means ± SE for 150 crypts from a total of three mice per group. **P<0.01 (Student's *t* test). **D**: Paraffin sections of the ileum from control or Shp2 CKO mice at 3 to 4 weeks of age were subjected to in situ hybridization analysis of Olfm4 mRNA. Representative images are shown in the left panel. Scale bar, 50 μm. The number of Olfm4 mRNA–positive cells per crypt was determined (right panel). Data are means ± SE for 60 crypts from a total of two mice per group. N.S., not significant (Student's *t* test).

We next examined the impact of IEC-specific Shp2 ablation on the numbers of absorptive enterocytes, mucin-secreting goblet cells, and antimicrobial peptide–producing Paneth cells in the intestinal epithelium. The number of absorptive enterocytes, which were identified on the basis of their morphology in tissue sections stained with a mAb to β-catenin [8], was markedly reduced in the ileum of Shp2 CKO mice at 3 to 4 weeks of age compared with that for control mice (**Fig. 4A**). We were not able to determine the precise number of absorptive enterocytes in the colon of Shp2 CKO mice because of their severe colitis. In addition, the number of mucin 2–positive goblet cells was also reduced in the ileum as well as the colon of Shp2 CKO mice at 3 weeks of age (**Fig. 4B**). We were again unable to determine the precise number of goblet cells in the colon of Shp2 CKO mice as a result of their severe colitis. In contrast, the number of lysozyme-positive Paneth cells was slightly increased in the ileum of Shp2 CKO mice at 3 weeks of age (**Fig. 4C**). These results thus suggested that Shp2 is important for regulation of the numbers of absorptive enterocytes and goblet cells in the mouse intestine.

We next investigated whether Shp2 regulates the size of the intestinal stem cell population, for which Olfm4 is a marker [24]. However, the number of Olfm4 mRNA–positive cells in crypts of the ileum did not differ between Shp2 CKO and control mice at 3 to 4 weeks of age (**Fig. 4D**).

### Impaired migration of IECs in the ileum of Shp2 CKO mice

**Figure 5:**
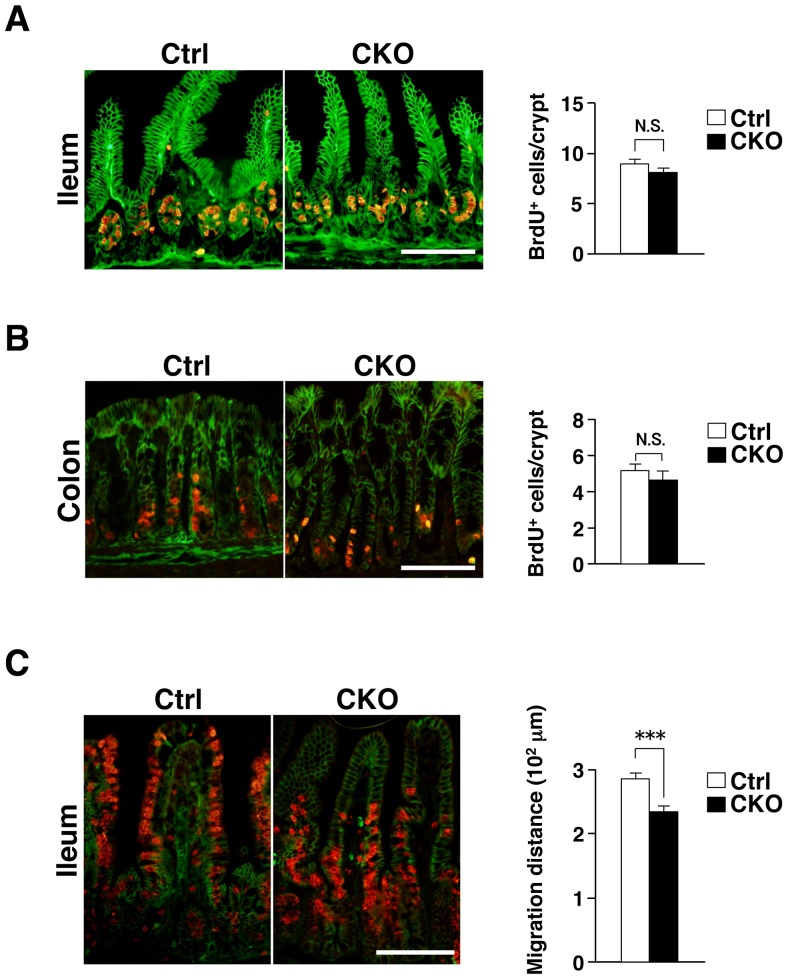
Impaired migration of IECs along the crypt-villus axis in the ileum of Shp2 CKO mice. **A**: Frozen sections of the ileum from 3- to 4-week-old control or Shp2 CKO mice at 2 h after BrdU injection were immunostained with mAbs to BrdU (red) and to β-catenin (green). Representative images are shown in the left panel. The number of BrdU-positive cells per crypt was determined (right panel). Data are means ± SE for 54 (control) or 77 (Shp2 CKO) crypts from a total of three (control) or four (Shp2 CKO) mice per group. N.S., not significant (Student's *t* test). **B**: Frozen sections of the colon from 3- to 4-week-old control or Shp2 CKO mice at 2 h after BrdU injection were immunostained as in **A**. The number of BrdU-positive cells per crypt was determined. Quantitative data are means ± SE for 54 (control) or 47 (Shp2 CKO) crypts from a total of three mice per group. N.S., not significant (Wilcoxon rank-sum test). **C**: Frozen sections of the ileum from 3- to 4-week-old control or Shp2 CKO mice at 2 days after BrdU injection were immunostained as in **A**. The distance from the crypt base to the farthest migrated BrdU-positive cells was measured. Quantitative data are means ± SE for 40 (control) or 48 (Shp2 CKO) villi from a total of three mice per group. ***P<0.0001 (Student's *t* test). All scale bars are 100 μm.

Given the role of Shp2 in promotion of cell growth or survival [10], we next examined the incorporation of BrdU into IECs as well as the turnover of BrdU-labeled IECs in Shp2 CKO mice at 3 to 4 weeks of age. At 2 h after BrdU injection, the number of BrdU-positive IECs in crypts of the ileum (**Fig. 5A**) or colon (**Fig. 5B**) from Shp2 CKO mice was similar to that for control mice. At 2 days after BrdU injection, most BrdU-positive IECs in the ileum of control mice had reached the middle region or top of villi (**Fig. 5C**). In contrast, such migration of BrdU-positive IECs along the crypt-villus axis was markedly delayed in Shp2 CKO mice (**Fig. 5C**). These results thus suggested that Shp2 is dispensable for the proliferation of IECs, in particular for that of TA cells, in crypts of the ileum or colon, but that it is required for migration of IECs along the crypt-villus axis in the ileum.

Absorptive enterocytes have a short life span (∼5 days), being released into the gut lumen after they have migrated to the tip of villi. This elimination of IECs is thought to be triggered, at least in part, by spontaneous apoptosis [3]. To investigate whether the marked reduction in the numbers of absorptive enterocytes and goblet cells apparent in the intestine of Shp2 CKO mice might be attributable to an increased frequency of apoptosis, we performed immunostaining with antibodies to the cleaved form of caspase-3. However, the number of cleaved caspase-3–positive IECs in the ileum or colon of Shp2 CKO mice did not differ significantly from that for control mice (**Fig. S2**).

### Impaired development of intestinal organoids from Shp2 CKO mice

**Figure 6:**
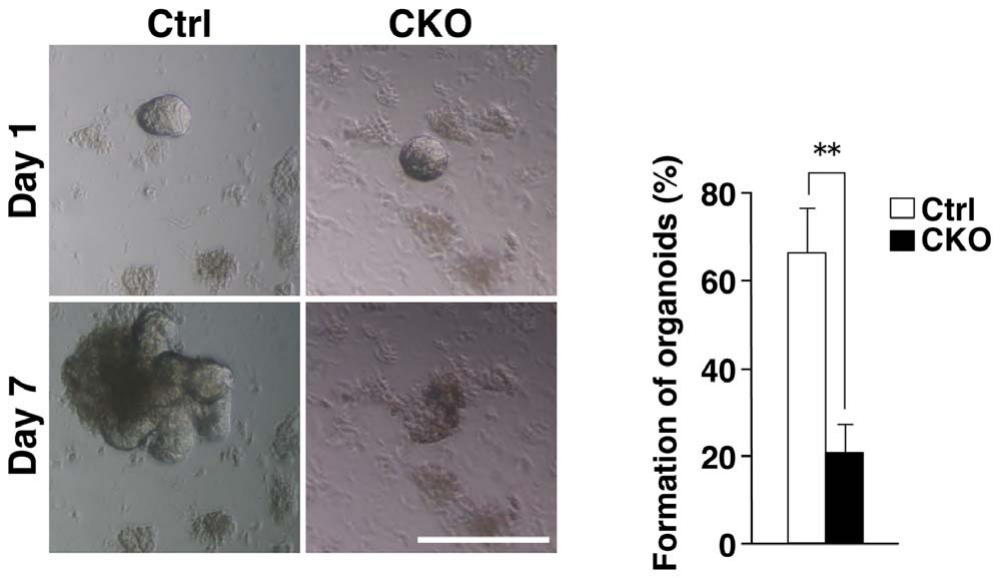
Impaired development of intestinal organoids from Shp2 CKO mice. Representative images of intestinal organoids derived from the jejunum of control or Shp2 CKO mice at 1 and 7 days after plating are shown in the left panel. Scale bar, 100 μm. The number of intestinal organoids with a crypt-villus structure at 7 days after plating was determined as a percentage of those with a spherelike morphology (diameter of >30 μm) at 1 day (right panel). Data are means ± SE for a total of 151 (control) or 128 (Shp2 CKO) organoids in four independent experiments. **P<0.01 (Student's *t* test).

The effect of Shp2 ablation in IECs on the development of villus structure from isolated crypts was investigated with the use of intestinal crypt-villus organoids [5]. The development of intestinal organoids from Shp2 CKO mice was found to be markedly impaired compared with that for control mice (**Fig. 6**). One day after seeding of isolated crypts, the morphology of the cultured crypts from Shp2 CKO mice was indistinguishable from that for control mice. However, 7 days after seeding, most of the crypts isolated from Shp2 CKO mice had failed to develop into intestinal organoids; they instead shrank or became cell debris. Quantitative analysis revealed that, whereas 66.6±9.9% of crypts from control mice developed into intestinal organoids, only 21.1±6.2% of those from Shp2 CKO mice did so. These results thus suggested that Shp2 is essential for efficient formation of intestinal organoids from isolated crypts.

### Expression of activated K-Ras in IECs protects Shp2 CKO mice against colitis

**Figure 7:**
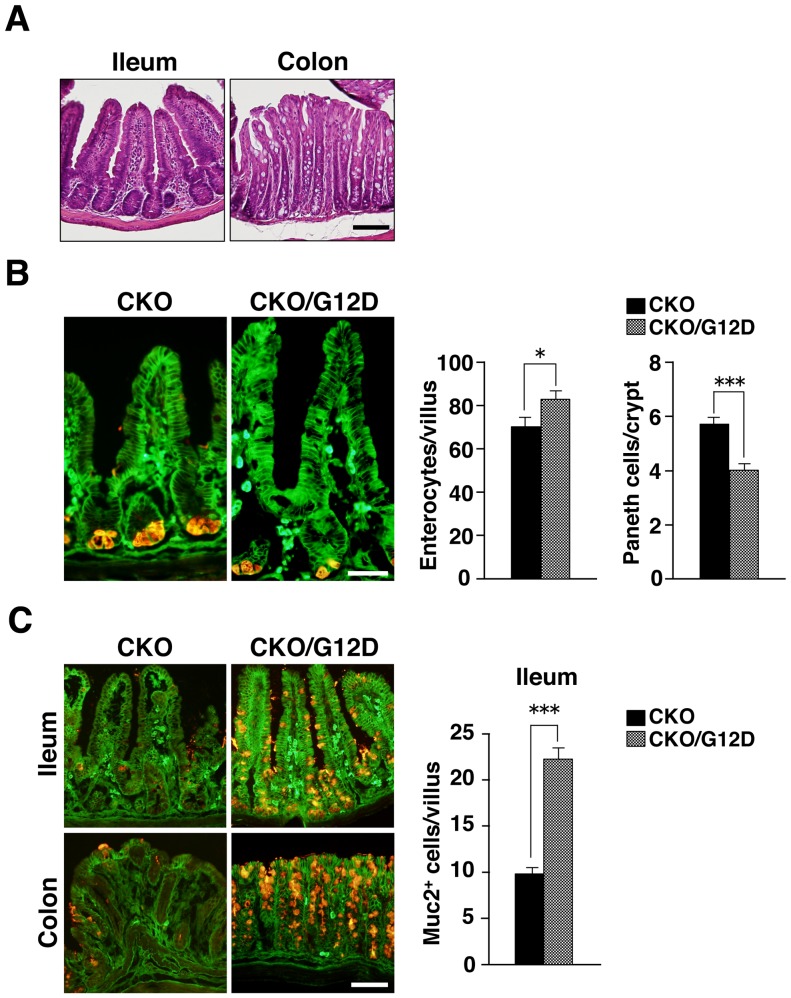
Expression of activated K-Ras in IECs protects Shp2 CKO mice against colitis. **A**: Hematoxylin-eosin staining of the ileum and colon from Shp2 CKO;*LSL-Kras G12D* mice at 4.5 weeks of age. Images are representative of those from three mice. Scale bar, 100 μm. **B**: Frozen sections of the ileum and colon from Shp2 CKO;*LSL-Kras G12D* (CKO/G12D) mice and their Shp2 CKO littermates at 4.5 weeks of age were immunostained with antibodies to lysozyme (red) and to β-catenin (green). Representative images are shown in the left panel. Scale bar, 100 μm. The middle panel shows the number of β-catenin –positive absorptive enterocytes per villus of the ileum. Data are means ± SE for 14 (CKO/G12D) or 10 (CKO) villi from a total of three (CKO/G12D) or two (CKO) mice per group. *P<0.05 (Student's *t* test). The right panel shows lysozyme-positive Paneth cells per crypt of the ileum. Data are means ± SE for 60 (CKO/G12D) or 43 (CKO) crypts from a total of three (CKO/G12D) or two (CKO) mice per group. ***P<0.0001 (Student's *t* test). **C**: Frozen sections of the ileum and colon from Shp2 CKO;*LSL-Kras G12D* (CKO/G12D) mice and their Shp2 CKO littermates at 4.5 weeks of age were immunostained with antibodies to mucin 2 (red) and to β-catenin (green). Representative images are shown in the left panel. Scale bar, 100 μm. The number of mucin 2–positive goblet cells per villus of the ileum was determined (right panel). Data are means ± SE for 30 (CKO/G12D) or 21 (CKO) villi from a total of three (CKO/G12D) or two (CKO) mice per group. ***P<0.0001 (Welch's *t* test).

Ras is an essential component of the signaling pathway by which growth factors stimulate cell proliferation, and the PTP activity of Shp2 is thought to regulate an upstream element necessary for Ras activation [10], [11]. The phenotypes of Shp2 CKO mice were thus likely to be attributable at least in part to impairment of the activation of Ras in IECs. To examine this notion, we crossed Shp2 CKO mice with *LSL-Kras G12D* mice [17], which harbor a gene for an activated form of K-Ras (K-Ras^G12D^). The crossing of Shp2 CKO (*Ptpn11* ^fl/fl^;villin-*cre*) mice with *LSL-Kras G12D* mice results in removal of translational stop elements by Cre-mediated recombination and consequent expression of the K-Ras^G12D^ gene under the control of its endogenous regulatory elements in an IEC-specific manner. Shp2 CKO;*LSL-Kras G12D* mice were born apparently healthy and phenotypically indistinguishable from their Shp2 CKO littermates (data not shown). Whereas Shp2 CKO mice were already sick at 4 weeks of age (**Fig. 2**), Shp2 CKO;*LSL-Kras G12D* mice (male or female) remained apparently healthy. Indeed, the body weight of Shp2 CKO;*LSL-Kras G12D* mice at 4 weeks of age was markedly greater than that of their Shp2 CKO littermates (**Table 1**), being similar to that of control mice (**Fig. 2A**). In addition, none of the Shp2 CKO;*LSL-Kras G12D* mice examined manifested any sign of colitis at or had died by 4.5 weeks of age (**Table 1**). Consistent with these findings, histological analysis of the ileum or colon from Shp2 CKO;*LSL-Kras G12D* mice revealed no sign of inflammation (**Fig. 7A**). These results thus suggested that expression of an active form of K-Ras in IECs protected Shp2 CKO mice from the development of colitis. We also found that the number of β-catenin –positive absorptive enterocytes was increased and that of Paneth cells was reduced in the ileum of Shp2 CKO;*LSL-Kras G12D* mice compared with those for Shp2 CKO mice at 4.5 weeks of age (**Fig. 7B**). In addition, we found that the number of mucin 2–positive goblet cells in the ileum or colon of Shp2 CKO;*LSL-Kras G12D* mice at 4.5 weeks of age was markedly greater than that for Shp2 CKO mice (**Fig. 7C**). The number of mucin 2–positive goblet cells in the ileum of Shp2 CKO;*LSL-Kras G12D* mice (22±1 per villus) was similar to or even slightly greater than that apparent in control mice (18 1 per villus) (**Fig. 4B** **)**. Together, these findings suggested that crossing of Shp2 CKO mice with *LSL-Kras G12D* mice restores the phenotypes of the former animals to normal.

**Table 1:** Phenotypes of three Shp2 CKO;*LSL-Kras G12D* mice and three Shp2 CKO littermates at 4.5 weeks of age.

Genotype	Sex	Body weight (g)	Disease activity index
CKO	Male	5.8	Dead
CKO	Male	7.5	6
CKO	Female	7.6	2
CKO/*Kras G12D*	Male	9.2	0
CKO/*Kras G12D*	Female	10.3	0
CKO/*Kras G12D*	Female	11.8	0

## Discussion

We have here shown that mice lacking Shp2 specifically in IECs develop severe colitis. The numbers of absorptive enterocytes and goblet cells were markedly reduced in the small intestine and colon of the mutant mice compared with those for control animals. Moreover, the development of intestinal organoids from isolated crypts of the Shp2 CKO mice was impaired. Shp2 is thought to be indispensable for activation of the Ras-MAPK signaling pathway and thereby for the promotion of cell proliferation and differentiation [10], [11], [25]. Shp2 in the nucleus is also important for activation of Wnt signaling [26], which is a key regulator of the proliferation and differentiation of IECs [2]. However, the colitis as well as the reduction in the number of absorptive enterocytes and goblet cells in the ileum of Shp2 CKO mice were prevented by expression of an activated form of K-Ras in IECs. Our present results thus indicate that Shp2 in IECs is important for protection against colitis as well as for homeostatic regulation of absorptive enterocytes and goblet cells, and that these functions of Shp2 are mediated through activation of the Ras-MAPK signaling pathway. The development of intestinal organoids essentially requires epidermal growth factor, which promotes IEC growth by activation of Ras-MAPK signalling, in the culture medium [5]. Thus, the impaired development of organoids from isolated crypts of the Shp2 CKO mice is most likely attributable to the impaired effect of epidermal growth factor on organoid development. Moreover, such impaired development of organoids in the culture system is well consistent with the IEC phenotype of Shp2 CKO mice such as reduced numbers of absorptive enterocytes and goblet cells, two major cell populations in IECs.

Shp2 is indispensable for embryogenesis at the early stages of gastrulation and mesoderm patterning [14]. In addition, conditional ablation of Shp2 in hematopoietic stem cells induces bone marrow aplasia [27], suggesting that Shp2 is essential for hematopoiesis from stem cells in the bone marrow. Given that Cre recombinase is expressed throughout the presumptive intestine of *villin-cre* mice by 14.5 days post coitum [18], ablation of Shp2 in the embryonic intestinal epithelium likely occurs in our Shp2 CKO mice. It is therefore of note that Shp2 CKO mice were born apparently healthy and that the overall development and morphology of the intestinal epithelium in these animals appeared almost normal up to 2 to 3 weeks of age (data not shown). Indeed, the population of Olfm4 mRNA–positive stem cells and BrdU incorporation into TA cells in crypts were not significantly affected in the small intestine of Shp2 CKO mice compared with control mice. Unlike the hematopoietic cell system, the Shp2-Ras signaling pathway thus does not appear to play a key role in the homeostasis of stem cells or in TA cell proliferation in the intestinal epithelium. In contrast, the Wnt signaling pathway is thought to be required for maintenance of intestinal stem cells as well as for the proliferation of IECs generated from these stem cells [2]. Indeed, ablation of the transcription factor Tcf-4, which is essential for operation of the Wnt signaling pathway in IECs, was found to result in marked defects in the development of intestinal villi, including the complete absence of the intestinal progenitor compartment [28].

Our results indicate that the Shp2-Ras signaling pathway is important for the development of mature IECs, in particular for that of absorptive enterocytes and goblet cells. The number of absorptive enterocytes in the small intestine was markedly reduced in Shp2 CKO mice. In addition, the number of goblet cells was also reduced in both the small intestine and colon of Shp2 CKO mice. Consistent with this finding, activation of K-Ras in IECs was previously shown to result in hyperplasia of the intestinal epithelium (suggesting an increase of absorptive enterocytes) as well as a marked increase in the number of goblet cells [29], [30]. Indeed, the reduction in the numbers of absorptive enterocytes and goblet cells in the ileum of Shp2 CKO mice was prevented by expression of an activated form of K-Ras in IECs. Thus, the Shp2-Ras signaling pathway is indeed important for the development of absorptive enterocytes and goblet cells. In contrast, Shp2 CKO mice manifested a significant increase in the number of Paneth cells in crypts. Conversely, we also showed that such increased number of Paneth cells was prevented by expression of an activated form of K-Ras in IECs. It was also showed that activation of K-Ras in IECs resulted in a marked reduction in the number of Paneth cells [30]. Thus, the Shp2-Ras signaling likely participates in negative regulation of the Paneth cell population.

The etiology of colitis in Shp2 CKO mice remains to be fully characterized. Goblet cells in the intestinal epithelium are thought to form a mucosal barrier by secreting mucus and thereby to protect against the development of intestinal inflammation [31]. Mucin 2 is the most abundant mucin produced by goblet cells, and deletion or mutation of the mucin 2 gene in mice results in the spontaneous development of colitis [32], [33]. The development of intestinal inflammation in Shp2 CKO mice is therefore most likely attributable to the marked reduction in the number of goblet cells in the intestine. Absorptive enterocytes are most abundant IECs and they form a physiological barrier against the intestinal microflora. We showed that the number of absorptive enterocytes was markedly reduced in Shp2 CKO mice, suggesting that the impairment of the physiological barrier provided by the intestinal epithelium likely occurs in Shp2 CKO mice. During the course of the present study, Coulombe et al. also showed that ablation of Shp2 in IECs resulted in the spontaneous development of colitis [34]. They also showed that expression of tight junctional proteins such as claudin was markedly reduced in the colon of their mutant mice, resulting in an increase in intestinal permeability [34]. Collectively, a defect in the physical barrier provided by the intestinal epithelium is likely a contributing factor to the development of colitis in Shp2 CKO mice. The number of Paneth cells was slightly increased in the ileum of Shp2 CKO mice. Paneth cells produce antimicrobial peptide such as α- or β-defensin, which are thought to be important for preventing the growth of pathogenic microbes [35], [36]. Thus, the increase of Paneth cells unlikely participates in the development of colitis in Shp2 CKO mice. The prevention of epithelial cell death by conditional ablation of caspase-8 in mouse IECs was also found to induce intestinal inflammation [37], suggesting that proper turnover of these cells is also important for homeostasis of intestinal immunity. In the present study, we found that IEC-specific ablation of Shp2 resulted in impaired migration of IECs along the crypt-villus axis. A delayed turnover of IECs may thus also contribute to intestinal inflammation in Shp2 CKO mice.

In summary, we have shown that Shp2 is necessary for homeostasis of IECs, in particular for that of absorptive enterocytes and goblet cells, as well as for protection against colitis. These functions of Shp2 are likely mediated by activation of Ras. Further investigation will be required, however, to clarify the molecular mechanism by which Shp2 in IECs regulates intestinal immunity and protects against colitis.

## Supporting Information

Figure S1 **Specific expression of β-galactosidase in the ileum and colon of R26R;villin-cre mice.** Frozen sections of the ileum or colon from adult *R26R*;villin-*cre* mice were stained for β-galactosidase activity (blue). Scale bar, 100 μm.
(TIFF)

Figure S2 **Lack of effect of Shp2 ablation on the number of apoptotic IECs in the ileum or colon.** Frozen sections of the ileum (**A**) or colon (**B**) from control or Shp2 CKO mice at 3 weeks of age were immunostained with antibodies to cleaved caspase-3 (red) and to β-catenin (green). Representative images are shown in the left panels. Scale bars, 100 μm. The number of cleaved caspase-3–positive cells per 10 villi in the ileum or 10 intestinal glands in the colon was determined (right panels). Data are means ± SE for 104 (control) or 118 (Shp2 KO) villi of the ileum and for 95 (control) or 92 (Shp2 CKO) intestinal glands of the colon from a total of two mice per group. N.S., not significant (Wilcoxon rank-sum test).
(TIFF)
