# Supplementary figures and images for: Role of the Protein Tyrosine Phosphatase Shp2 in Homeostasis of the Intestinal Epithelium
Source: PLoS One. 2014 Mar 27;9(3):e92904. doi: 10.1371/journal.pone.0092904 (PMC3968040; doi:10.1371/journal.pone.0092904)

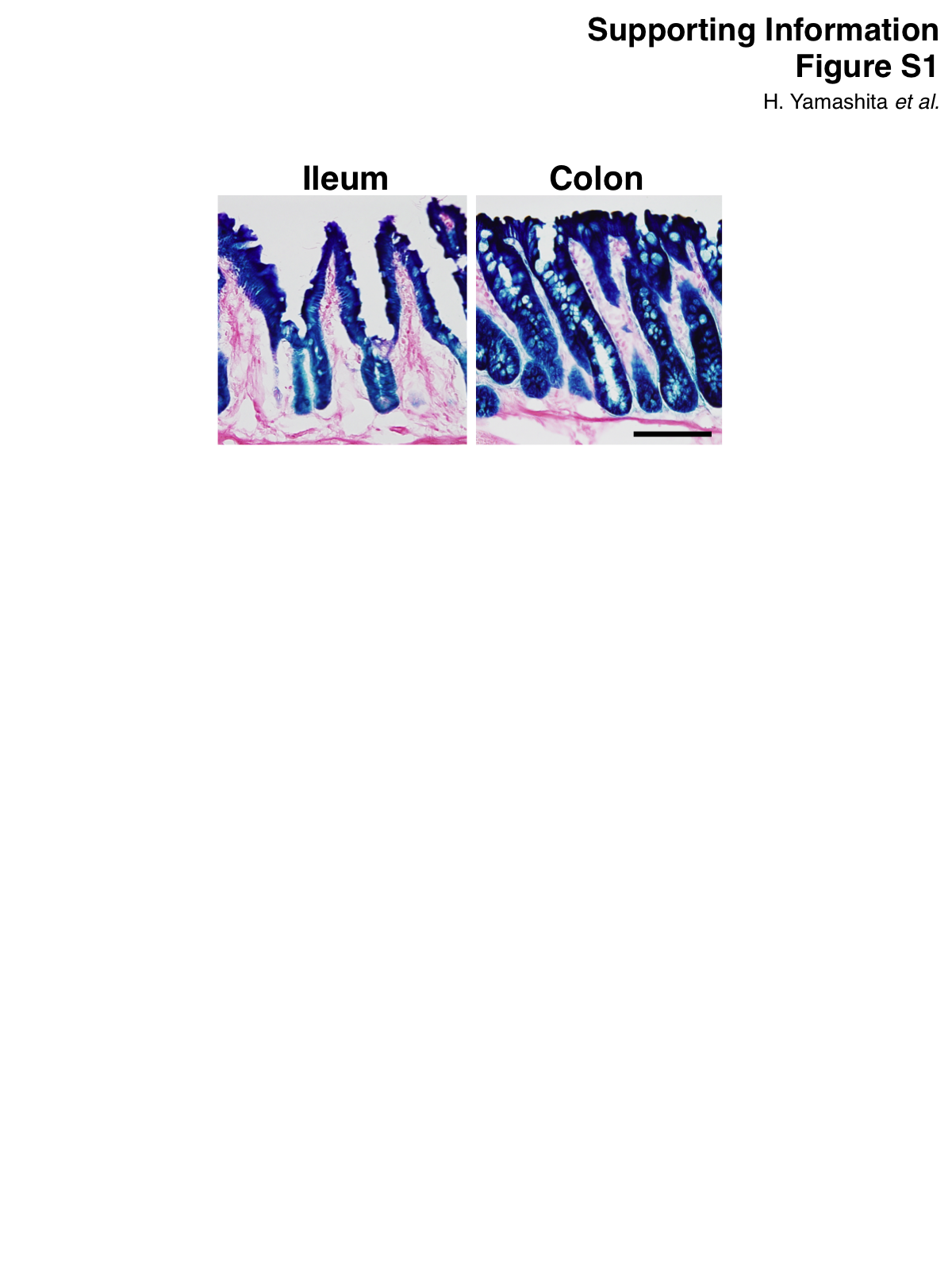

Supplement: Figure S1 — Specific expression of β-galactosidase in the ileum and colon of R26R;villin-cre mice. Frozen sections of the ileum or colon from adult R26R;villin-cre mice were stained for β-galactosidase activity (blue). Scale bar, 100 μm. (TIFF) [file pone.0092904.s001.tiff]

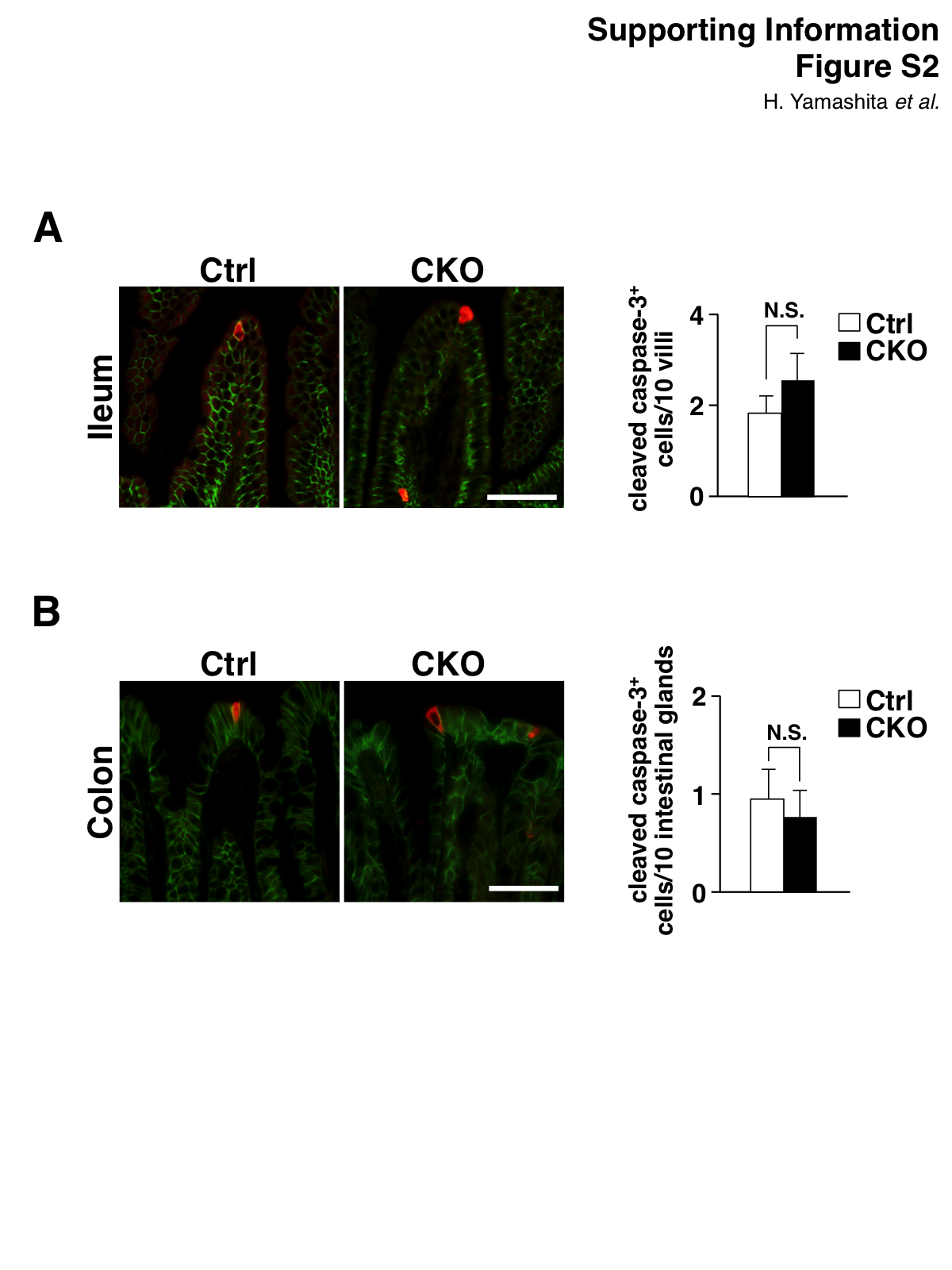

Supplement: Figure S2 — Lack of effect of Shp2 ablation on the number of apoptotic IECs in the ileum or colon. Frozen sections of the ileum (A) or colon (B) from control or Shp2 CKO mice at 3 weeks of age were immunostained with antibodies to cleaved caspase-3 (red) and to β-catenin (green). Representative images are shown in the left panels. Scale bars, 100 μm. The number of cleaved caspase-3–positive cells per 10 villi in the ileum or 10 intestinal glands in the colon was determined (right panels). Data are means ± SE for 104 (control) or 118 (Shp2 KO) villi of the ileum and for 95 (control) or 92 (Shp2 CKO) intestinal glands of the colon from a total of two mice per group. N.S., not significant (Wilcoxon rank-sum test). (TIFF) [file pone.0092904.s002.tiff]
